# Retraction: Overexpression of PCDH8 inhibits proliferation and invasion, and induces apoptosis in papillary thyroid cancer cells

**DOI:** 10.1039/d1ra90011k

**Published:** 2021-01-20

**Authors:** Laura Fisher

**Affiliations:** Royal Society of Chemistry Thomas Graham House, Science Park, Milton Road Cambridge CB4 0WF UK advances-rsc@rsc.org

## Abstract

Retraction of ‘Overexpression of PCDH8 inhibits proliferation and invasion, and induces apoptosis in papillary thyroid cancer cells’ by Liang Chang *et al.*, *RSC Adv.*, 2018, **8**, 18030–18037, DOI: 10.1039/C8RA02291G.

The Royal Society of Chemistry hereby wholly retracts this *RSC Advances* article due to concerns with the reliability of the data. The images in the article were screened by an image integrity expert who identified instances of manipulation of western blots, which undermines the reliability of all the western blots presented in the article.

In Fig. 1D, the PCDH8 band shows signs of image manipulation. The bottom edge of the second band is missing and there are also repeating patterns in the background. In addition, in the GAPDH control panel, the 2^nd^ and 4^th^ bands are duplicates.

The authors contacted the Editor asking to retract the article because they found that PCDH8 has no effect on papillary thyroid cancer cell apoptosis. The authors informed us that they performed this assay again and found that their prior results were wrong.

Given the significance of the concerns about the validity of the data in the article, the findings presented in this paper are not reliable.

The authors did not respond to any correspondence regarding the wording of the retraction notice.

Signed: Laura Fisher, Executive Editor, *RSC Advances*

Date: 7^th^ January 2021

## Supplementary Material

